# Comprehensive Transcriptomic Analysis of VISTA in Acute Myeloid Leukemia: Insights into Its Prognostic Value

**DOI:** 10.3390/ijms232314885

**Published:** 2022-11-28

**Authors:** Simona Pagliuca, Carmelo Gurnari, Keman Zhang, Tariq Kewan, Waled Bahaj, Minako Mori, Ishani Nautiyal, Marie Thérèse Rubio, Francesca Ferraro, Jaroslaw P. Maciejewski, Li Wang, Valeria Visconte

**Affiliations:** 1 Translational Hematology and Oncology Research Department of Cleveland Clinic, Cleveland, OH 44106, USA; 2 Service d’hématologie, Hôpital Brabois, CHRU Nancy and CNRS UMR 7365 IMoPa, Biopôle de l’Université de Lorraine, 54500 Vandoeuvre les Nancy, France; 3 Department of Biomedicine and Prevention, University of Rome Tor Vergata, 00133 Rome, Italy; 4 Department of Molecular Medicine, Cleveland Clinic Lerner College of Medicine, Case Western Reserve University, Cleveland, OH 44195, USA; 5 Division of Oncology, Department of Medicine, Washington University School of Medicine in St. Louis, St. Louis, MO 63110, USA

**Keywords:** VISTA, AML immunotherapy, immune escape, immune checkpoint regulation, NPM1

## Abstract

The V-domain Ig suppressor of T-cell activation (VISTA) has been recognized as a critical negative regulator of antitumor immune response and is gaining growing interest as a potential pharmacological target in immunotherapy. This molecule is highly expressed in hematopoietic stem cells and myeloid compartment, and it has been found upmodulated in acute myeloid leukemia (AML). However, VISTA-associated immune features are relatively unexplored in myeloid malignancies. Herein, we aimed to explore whether this immune checkpoint regulator could play a role in the generation of an immune escape environment in AML patients. We characterized VISTA mRNA expression levels in leukemia cell lines and in large publicly available cohorts of specimens from bone marrow of healthy individuals and AML patients at diagnosis by deploying bulk and single-cell RNA sequencing. We also defined the correlations with leukemia-associated burden using results of whole-exome sequencing of AML samples at disease onset. We showed that VISTA expression linearly increased across the myeloid differentiation tree in normal hematopoiesis. Accordingly, its transcript was highly enriched in AML cell lines as well as in AML patients at diagnosis presenting with myelomonocytic and monocytic differentiation. A strong correlation was seen with *NPM1* mutations regardless of the presence of *FLT3* lesions. Furthermore, VISTA expression levels at baseline correlated with disease recurrence in patients with normal karyotype and *NPM1* mutations, a subgroup traditionally considered as favorable according to current diagnostic schemes. Indeed, when compared to patients with long-term remission (>5 years after standard chemotherapy regimens), cases relapsing within 2 years from diagnosis had increased VISTA expression in both leukemia and T cells. Our results suggest a rationale for developing VISTA-targeted therapeutic strategies to treat molecularly defined subgroups of AML patients to prevent disease recurrence and treatment resistance.

## 1. Introduction

The engagement of immune checkpoint receptors (ICRs), the physiological gatekeepers of immune responses, constitutes one of the milestones of cancer immune evasion [1]. The use of inhibitors targeting ICRs has revolutionized the therapeutic landscape of solid tumors [1]. However, early on, it became evident that the response and resistance to immunotherapies varied greatly among patients, cancer types, and specificities of ICR targeted [2,3]. Therefore, in the last decade increasing efforts have been made to identify the molecular and clinical underpinnings associated with response to immune checkpoint inhibition [3].

In contrast to their successful use in solid cancers, monoclonal antibodies targeting ICRs have yielded limited therapeutic efficacy in acute myeloid leukemia (AML) [4]. One could speculate that additional, nonredundant ICRs, specifically expressed in transformed leukemia cells and/or in AML-infiltrating immune cells, may represent better targets in this disease [5]. As a consequence, the identification and characterization of these AML-specific ICRs becomes critical for the design of effective AML-specific immunotherapies [6].

In this regard, modulation of VISTA signaling is gaining growing interest due to its expression on different subsets of immune and cancer cells, acting both as a ligand and as a receptor [7,8,9,10].

We and others have shown that, unlike other inhibitory ICRs expressed on activated T cells, VISTA is highly expressed on naïve T cells, as well as on hematopoietic stem cells (HSCs), myeloid progenitors, myeloid-derived suppressor cells (MDSCs), and antigen-presenting cells (APCs), including dendritic cells (DCs) and macrophages [11,12,13,14].

Mechanistically, VISTA expression on APCs and tumor cells hampers effective T-cell tumor reactions by reducing T-cell proliferation and cytokine production, as well as inducing the activation of Foxp3+ Tregs [9,10,11]. In addition to regulation of T-cell responses, earlier studies from our group revealed a novel function of VISTA in the modulation of the inflammatory responses of macrophages and monocytes [14]. Therefore, VISTA blockade may be considered an attractive strategy to finely tune antitumor immune control by (i) enhancing tumor-reactive T cell responses via regulating T-cell activation pathways; (ii) reducing MDSC abundance and suppressive function; (iii) controlling the type of inflammatory response (type I vs. type II) in the tumor microenvironment. In hematological malignancies, VISTA has been found upregulated in myeloid disorders such as leukemias with monocytic differentiation and in monocytic MDSCs of patients with AML [13,15]. Nevertheless, the expression patterns of VISTA remain still underexplored in these disorders. 

Through integrated genomic and transcriptomic analyses, we studied this pleiotropic ICR in normal and malignant hematopoiesis along with its association with mechanisms of immunoediting and genomic instability.

## 2. Results

### 2.1. Cartography of VISTA Expression across Normal Hematopoiesis and Hematological Cancer Cell Lines

**Figure 1:**
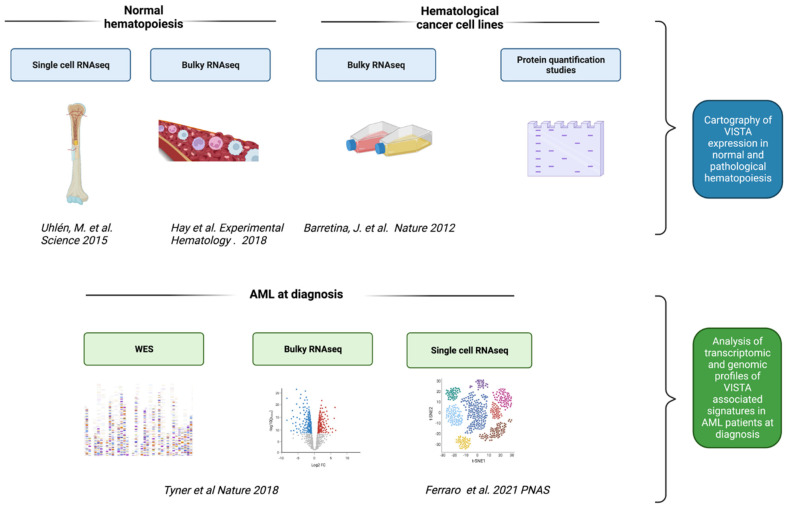
**Flow chart showing the study design**. We determined a cartography of VISTA expression across normal hematopoiesis stages and hematological cancer cell lines by integrating multiple transcriptomic data from public resources and protein quantification studies. Then, we analyzed the transcriptomic and genomic profiles of VISTA associated signatures in acute myeloid leukemia (AML) cases at diagnosis. Next, we analyzed single-cell RNAseq data from an independent cohort of patients with normal karyotype AML, treated homogeneously with standard induction and consolidation chemotherapy and with well-annotated remission data after treatment. We then compared phenotypic data to understand the interplay between AML and T cells with regard to VISTA expression. Parts of the figure were created with BioRender.com [5,16,17,18,19].

**Figure 2:**
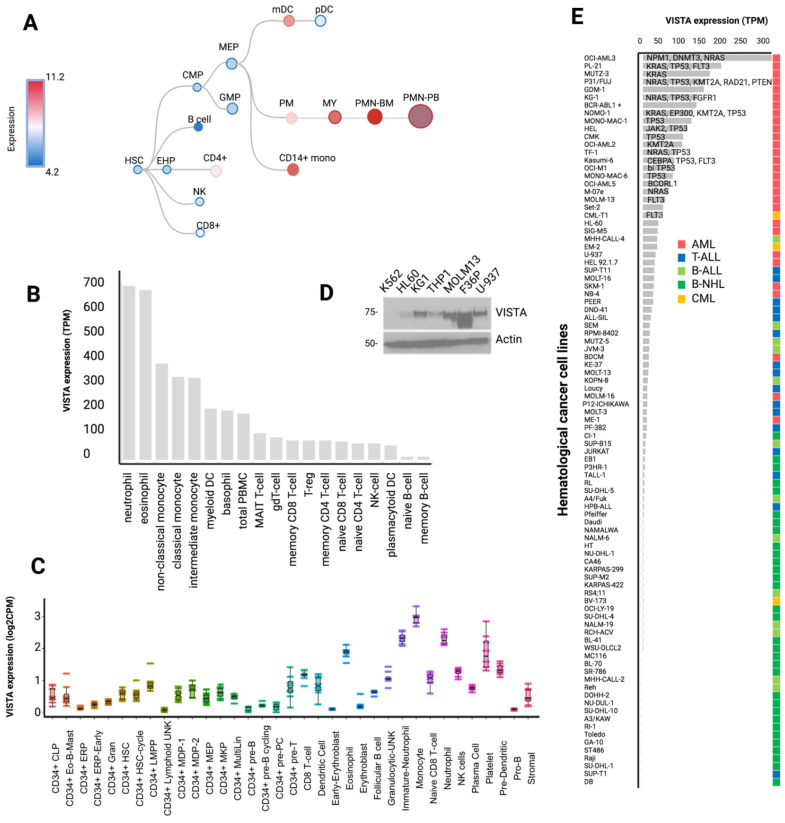
**VISTA transcriptomic profile in normal hematopoiesis and in hematological cancer cell lines.** (**A**) Vista differentiation tree across hematopoietic cell differentiation (data retrieved and adapted from https://servers.binf.ku.dk/bloodspot/, 28 January 2022). (**B**) Bar plot showing the distribution of VISTA expression values (as transcripts per million, TPM) across several mature peripheral blood cell populations. Raw data were retrieved from https://www.proteinatlas.org/about/download, 21 December 2021. (**C**) Box plot displaying the distribution of VISTA expression values (TPM) across 35 bone marrow cell types (raw data retrieved from http://www.altanalyze.org/ICGS/HCA/Viewer.php, 13 September 2021). (**D**) Western blot showing VISTA protein abundance in several hematological cell lines. (**E**) Bar plot of VISTA expression across hematological cancer cell lines (data retrieved from https://www.ebi.ac.uk/gxa/experiments/E-MTAB-2770/Downloads, 13 September 2021). The text indicates the name of mutated genes in each cell lines. Only representative cell lines are reported.

First, we determined a cartography of VISTA expression across normal hematopoiesis stages, peripheral blood cells, and hematological cancer cell lines (Figure 1) by integrating multiple transcriptomic data from public resources and protein quantification studies (see Section 3). We observed that VISTA expression linearly increased across the myeloid differentiation tree, showing more abundant mRNA levels in monocytes and in differentiated polymorphonucleated cells (Figure 2A), particularly in neutrophils and eosinophils (Figure 2B). In contrast, very low to null levels of expression were observed in the lymphoid compartment, including progenitors of committed B cells, as well as CD4 and CD8 T cells. The single-cell transcriptomic profile of normal donor bone marrow specimens was consistent with this expression pattern (Figure 2C, Table S1) [16]. Similarly, the analysis of a number of human leukemia and lymphoma cell lines, by means of either immunoblotting (Figure 2D) or RNASeq [17] (Figure 2E), showed increased VISTA expression in myeloid neoplasia as compared to lymphoid subsets (Table S2). Specifically, the highest level of VISTA transcript was found in OCI-AML3 (*NPM1-*positive cell line) and in PL-21 and MUTZ-3 cell lines (all carrying a *KRAS* mutation; Figure 2E).

### 2.2. VISTA Expression Profile in AML

Upon assessing VISTA transcriptomic profiles and establishing their relationships with AML, we sought to understand VISTA expression in association with clinical–biological features of AML. To that end, we analyzed 285 AML cases at diagnosis from a well-annotated publicly available cohort of patients (Table S3) [18]. VISTA was found to be significantly upregulated in AML at diagnosis as compared to healthy controls (*p* = 0.0002, Figure S1A). While no significant difference was found across the European Leukemia Net (ELN) 2017 classification groups (Figure S1B), a higher VISTA expression was noted in morphological subtypes with myelomonocytic and monocytic differentiation, recapitulating the transcriptomic patterns observed in the normal stages of myelopoiesis (Figure 2A). Accordingly, *NPM1* mutations and *MLLT3–KTM2A* gene fusions were the genomic aberrations associated with higher VISTA expression (Figure 2A and Figure S1C,D), possibly due to a higher enrichment in classical M4 and M5 French–American–British (FAB) morphologic subgroups (Figure S1B). Specifically, *NPM1*-mutant patients displayed higher levels of VISTA transcripts compared to wildtype cases, regardless of the presence of *FLT3* internal tandem duplication (*FLT3*-ITD) (Figure 2B,C and Figure S2). Importantly, VISTA expression did not correlate with blast infiltration in bone marrow at diagnosis (Figure S1E), excluding the potential risk of interpretation bias in patients with higher disease burden. A weak correlation was instead observed with age at disease onset (Figure S1F,G), with younger patients presenting with lower VISTA levels at presentation.

Next, we examined whether VISTA expression may influence the transcriptional and phenotypic features of AML. We categorized our cohort in high (N = 139) and low (N = 146) VISTA expressors, according to the 50th percentile of VISTA mRNA levels in 21 healthy control specimens from the same dataset, and we performed a differential gene expression analysis between these two groups.

**Figure 3:**
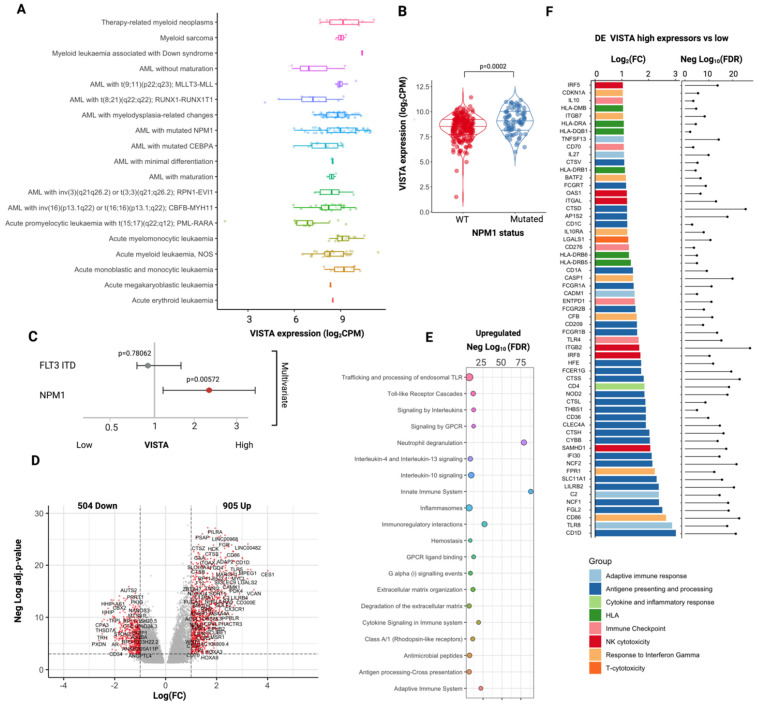
**VISTA transcriptomic profile in AML.** (**A**) Boxplots showing the distribution of VISTA expression (logarithmic count per million, logCPM) across several acute myeloid leukemia (AML) subtypes (Beat AML cohort). See Table S8 for pairwise comparisons. (**B**) Violin plots representing the differences in distribution of VISTA expression values in patients with *NPM1-*positive vs. *NPM1*-negative AML (data from bulk RNAseq samples). (**C**) Multivariate logistic regression model showing the independence of VISTA from *FLT3* mutational status in *NPM1* cases. Distribution of VISTA expression values according to the different AML FAB subtypes. (**D**) Volcano plot representing the differential expression analysis of genes up- and down-regulated in VISTA high vs. low expressors. (**E**) Selected reactome pathways enriched in the gene sets upregulated in VISTA high vs. low expressors (negative log_10_ of the adjusted *p*-value—Benjamini–Hochberg correction, gene set enrichment analysis). (**F**) Selected genes involved in immune responses upregulated in the analysis of VISTA high vs. low expressors (bar plot representing log_2_ fold change and lollipop plot showing the negative log_10_ of the adjusted *p*-value—Benjamini–Hochberg correction).

Our analysis showed that tumor tissues with higher VISTA expression were enriched in genes involved in immune activation with upregulation of antigen presentation and processing pathways, cytokine and interleukin signaling, Toll-like receptor cascade, NK cytotoxicity, and response to interferon (Figure 3D–F). Specifically, genes involved in Th1 and Th2 responses, but not T cytotoxic-related genes, were significantly enriched in high VISTA expressors. Consistent with the upregulated CD4 T-cell responses, most class II HLA genes were overexpressed in this subgroup of patients, whereas only a modest upregulation of class I HLA loci was present (Tables S4 and S5). A linear correlation existed between VISTA mRNA expression and most HLA gene transcripts (Figure S3A).

### 2.3. VISTA Expression Correlates with a Lower Mutational Burden and with a Specific Myeloid Landscape

The significant upregulation of CD4 Th1 and Th2 response signatures in VISTA high expressors indicates an immune activation directed toward class II HLA-restricted tumor antigens and, therefore, a potential vulnerability of AML cells via a mechanism of immune editing. To further explore this aspect, we analyzed the correlation between VISTA expression and both the mutational burden and the landscape of these two groups of patients. We found that higher VISTA expression was correlated, on one hand, with a significantly lower number of somatic mutations (mean = 6 vs. 10, *p* = 0.00033, linear regression in Figure S1H) and, on the other hand, with a specific landscape of myeloid driver aberrations. Similarly to what we noticed in myeloid leukemia cell lines, high VISTA expressors had indeed an increased prevalence of aberrations in *NPM1* (odds ratio [OR] 2.1 [95% confidence interval (CI): 1.19–4.2], *p* = 0.001) and *KRAS* (OR: 4.1 [95% CI: 1.2–22], *p* = 0.031), whereas lower expressors displayed a higher frequency of somatic alterations in *CEBPA* (OR: 0.28 [95% CI: 0.10–0.80], *p* = 0.041), *RUNX1* (OR: 0.45 [95% CI: 0.21–0.99], *p*= 0.012), and *PHF6* (OR: 0.09[95% CI: 0.01–0.74], *p* = 0.0057, Table S6, Figure S2). Interestingly, linear regression models between VISTA gene transcripts and NPM1 and KRAS mRNAs showed results consistent with these molecular associations (Figure S3B,C).

### 2.4. Leukemic T-Cell VISTA Interactions in NPM1 Mutated AML

**Figure 4:**
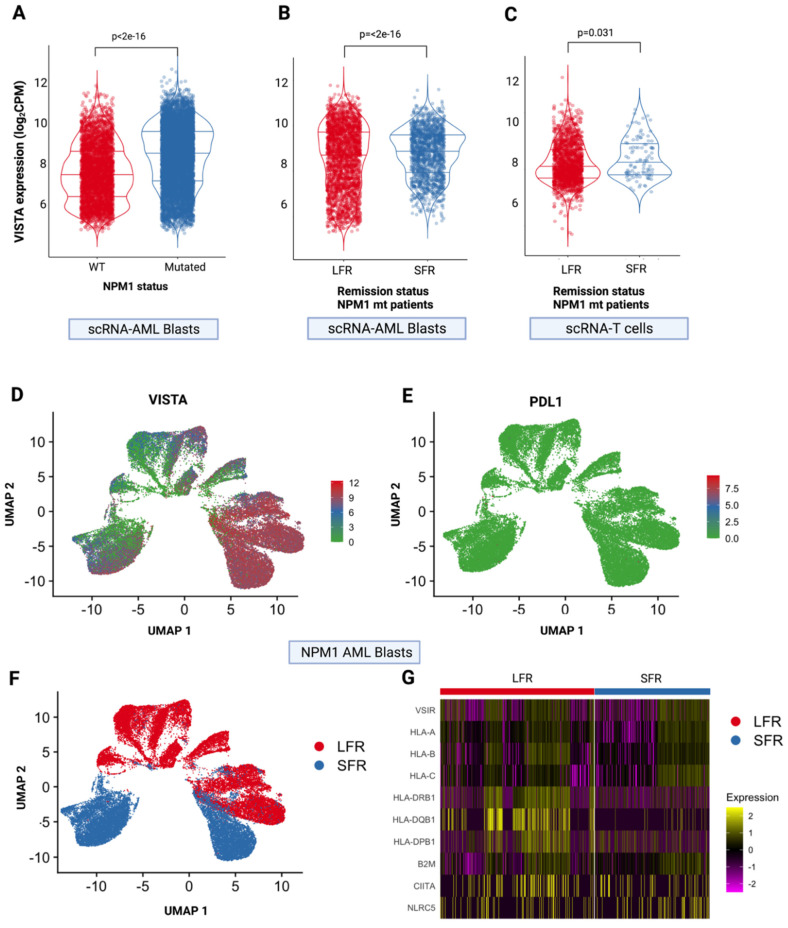
**VISTA expression at single cell level.** (**A**) VISTA expression in single-cell RNAseq samples from isolated AML blasts according to *NPM1* mutational status. (**B**) VISTA expression in single-cell RNAseq samples of AML blasts from patients with *NPM1* mutations according to the length of first remission (long, *LFR*, >5 years vs. short*, SFR*, <2 years). (**C**) VISTA expression in single-cell RNAseq samples of T cells from patients with *NPM1* mutations according to first remission status (long, *LFR*, >5 years vs. standard*, SFR*, <2 years). (**D**) Uniform manifold approximation and projection for dimension reduction (UMAP) of VISTA and (**E**) PDL-1 expression in AML cells from *NPM1*-mutated patients (N = 9 single-cell RNAseq samples). Each dot represents a cell; gray dots indicate low or null expression. The purple gradient indicates the intensity of each gene expression (VISTA on the left and PDL1 on the right). (**F**) UMAP showing the distribution of AML cells in LFS and SFR groups. (**G**) Heatmap showing the difference in expression of VISTA, HLA genes and HLA class I and II transactivators between LFR and SFR patients.

To determine whether VISTA may play a role in driving immune evasion in *NPM1*-mutant patients and to further explore the VISTA-mediated interplay between AML cells and T cells, we analyzed single-cell RNA-sequencing from matched patients with normal-karyotype AML, who underwent standard induction and consolidation chemotherapy and had long (LFR: absence of relapse over a follow-up period of >5 years) versus short remissions (SFR: relapse within 2 years) [5]. We first confirmed VISTA overexpression in AML cells of *NPM1*-mutant vs. wildtype cases (Figure 4A), which corroborated our previous results. After restricting the analysis to the *NPM1*-mutant group, we observed that VISTA expression was significantly upregulated in both leukemic (*p* < 2 × 10^−16^, Figure 4B) and CD3+ cells (*p* = 0.031, Figure 4C, Table S7) in SFR when compared to LFR cases. 

When we analyzed the per-cell distribution of VISTA expression in *NPM1*-mutated samples, we found that it was homogeneously represented on leukemic cells, while other immune checkpoint ligands, namely, PD-L1, were virtually absent (Figure 4D,E). Uniform manifold approximation and projection for dimension reduction (UMAP) graphs showed that samples from SFR patients were those with a higher level of VISTA expression (Figure 4F).

Furthermore, at the single-cell level, it was interesting to notice that cells with higher VISTA expression in both SFR and LFR groups were associated with higher levels of class II HLA gene transcripts (Figure 4G), confirming the observations in bulk RNASeq analyses, although, due to the small sample size of this subgroup, this finding did not translate in a tangible difference in disease free survival (Figure S4).

## 3. Discussion

Upmodulation of inhibitory checkpoint molecules represents a common strategy adopted by cancer cells to evade immune surveillance. Our work describes the complex molecular landscape underpinning the dysregulation of VISTA, which has the potential to become a next-generation ICR target in AML.

The potential role of the VISTA blockade is supported by the specificity of this ICR for myeloid lineage and for morphological and molecular subtypes of AML as shown in the transcriptomic profile of cell lines and patients. The upregulation of VISTA on leukemic and T cells may play an important role in impairing immune surveillance mechanisms against AML cells, working as inhibitory feedback to counteract T effector cell control. The correlation among VISTA expression, *NPM1* mutation, and AML monocytic phenotypes confirms previous observations [15]. While its expression is associated with lower tumor burden, possibly mirroring the relatively homogeneous genomic makeup of *NPM1*-mutant AML, its overexpression in this traditionally favorable subgroup would indicate a higher propensity to immune suppression from AML cells, coupled with exhaustion of endogenous T cells. In such a case, increased VISTA expression would affect patient outcome by hampering the immunological control favoring leukemia relapse after chemotherapy. 

Despite recent progress, the outcomes of patients with AML relapsing after standard chemotherapy are still dismal with overall survival rates of about 30% at 5 years [20]. Thus, studies aiming at a better characterization of determinants of relapse represent an unmet clinical need in this disease. To that end, we show how VISTA expression may offer guidance in the early identification of cases at higher risk for future relapse, possibly helping in selecting patients in need of treatment intensification strategies. Indeed, our analyses point toward a possible role of VISTA in promoting immunosuppression following tumor-reactive T-cell activation, thereby driving immune escape and impairing clinical outcomes. 

The class II HLA-restricted T-cell selection may play a role in favoring the upmodulation of this ICR on leukemic cells as a feedback mechanism in response to the immunoediting. Nonetheless, it is possible that the particular genomic configuration of high VISTA-expressing cells may lead to a specific pattern of neoantigen presentation, thereby resulting in enhanced immunogenicity toward T helper responses.

It is noteworthy to highlight that, while VISTA is heavily overexpressed in AML cells, other negative ICRs are virtually absent at diagnosis. A possible explanation of this imbalanced distribution of immune checkpoint molecules on target cells is that VISTA represents an early regulator of immune responses, driving the reaction to immunoediting in the early disease phases. Additionally, it is possible that VISTA may play a role in myeloid differentiation, given the cell-specific topography of its expression across the hematopoietic tree. 

On the basis of these results, we conclude that VISTA overexpression on AML cells may represent an early feedback mechanism to counteract immune activation-mediated pressure in controlling tumor progression. Hence, we advocate for a redefinition of the role of VISTA as an early biomarker of immune escape in AML able to identify, at diagnosis, patients at higher risk for relapse. Our results provide a rationale for developing VISTA-targeted therapeutic strategies to treat molecularly defined subgroups of AML patients to prevent disease recurrence and treatment resistance. 

Future studies are warranted to explore the potential causative role of VISTA signaling in influencing the immune homeostasis inherent to cancer and the emergence of molecular resistances to standard treatments.

## 4. Methods and Materials

### 4.1. Study Overview

Taking advantage of publicly available resources, we performed multi-omics genetic and transcriptomic analyses to investigate the role of VISTA as an immune checkpoint target in AML (Figure 1).

We started from transcriptomic data available in normal donors and cancer cell lines to describe VISTA distribution across the hematopoietic differentiation hierarchy, across major blood and marrow cell populations and leukemia and lymphoma cells (Tables S1 and S2) [16,17,19].

We then proceeded with the investigation of its role in patients with AML at diagnosis. For this purpose, we used samples from The Beat AML master trial, the largest-to-date data repository of AML biospecimens accounting for 672 samples, including whole-exome data, RNA-seq, and ex vivo drug sensitivity data [18]. For clarity purposes, the clinical annotations for the subgroup of patients analyzed in this study are detailed in the Supplementary Materials (Tables S3 and S8). 

We then explored and compared at a single-cell RNA level, in both AML cells and T-cell fractions, the regulation of VISTA, the associated immune signatures, and its correlation with response to chemotherapy and remission duration in a cohort of AML patient with long-term follow-up [5].

Details on patient and sample selection, as well as on the sequencing and analytical workflows, are reported in the original studies [5,18].

### 4.2. Transcriptomic and Differential Expression Analysis

For all the analyses described in the study, normalized gene counts were retrieved from the original studies and open-source tools. For the description of VISTA expression in normal hematopoiesis, normalized transcripts per million (TPM) values were used. For AML samples, we used normalized logarithmic counts per million. Normalization procedures have been previously described [16,17,18,19]. We applied a post-normalization analytical pipeline to remove the batch effect and to filter out the non-expressed genes. R Bioconductor environment, Edge R, and DEseq2 packages were used for differential gene expression analysis of bulk RNAseq data [21]. The log_2_ fold-change (logFC) and likelihood test were calculated along with the false discovery rate (FDR), obtained using the Benjamini–Hochberg method for multiple testing correction [22]. Gene set enrichment analysis was performed via the Gsea-Msigdbv.7.4 software (http://www.gsea-msigdb.org/gsea/msigdb/annotate.jsp, downloaded on 22 September 2021) to identify the functional subsets of genes differentially expressed in High vs. Low VISTA expressors [23]. Overlaps with REACTOME gene sets were also explored. 

### 4.3. Single-Cell RNA Sequencing and Analysis

Single-cell AML specimens were sequenced and analyzed in Washington University (St. Louis, MO, USA). This cohort was recently described [5]; however, only a few selected specimens of the entire sequenced cohort were included in this study. Specifically, we selected samples from patients who presented at diagnosis with an *NPM1*-mutated favorable or intermediate-risk AML according to ELN 2017 in both long (greater than 5 years) and standard (less than or equal to 2 years) first remissions after chemotherapy groups [24]. Samples were preprocessed and sequenced as previously described [5]. Cellranger v3.1.0 platform was used for alignment, post-alignment processing, and gene annotation. Ensembl version 93 cellMatch [25] was used for cell population identification and annotation, while the Partek Flow software (build version 10.0.20.1231) was used to import gene/barcode matrices and lineage assignments. Seurat package was used for quality control assessment, filtering, normalization, differential analysis, and data visualization [26].

### 4.4. Genomic Analysis

Specimens from patients with leukemia at diagnosis recruited in The Beat AML master trial subjected to both whole-exome and transcriptomic sequencing were included in our study. Details on the genomic and transcriptomic sequencing platforms, as well as bioinformatic analysis for variant calling, were reported in the original study [18]. Only somatic calls derived from the VarScan variant calling algorithm were used for the purposes of our study. A total of 245 samples were analyzed for genomic–transcriptomic associations.

### 4.5. Western Blot Study

Cells (5 × 10^6^) were lysed in RIPA buffer supplied with 1× protease inhibitor cocktail (ThermoFisher Scientific, Waltham, MA, USA) on ice for 20 min. Ten micrograms of proteins were mixed with Laemmli sample buffer 4× plus reducing agent, boiled at 95 °C for 5 min. Samples were run using NuPAGE™ 4% to 12%, Bis-Tris (ThermoFisher Scientific). Proteins were transferred onto blotting membranes and incubated overnight at 4 °C with an anti-VISTA (D1L2G™) XP^®^ Rabbit monoclonal antibody (Cell Signaling, Danvers, MA, USA; Catalog#64953). An anti-actin monoclonal antibody was used for loading control.

### 4.6. Statistical Analyses

Median values, ranges, and percentages were used for the description of the continuous and categorical variables. For all relevant comparisons, a Wilcoxon matched pairs signed rank test at 95% CI was used. Fisher’s exact test was applied for independent group comparisons; in the case of testing more than two groups, a one-way ANOVA test was used. All *p*-values were two-sided, and values less than 0.05 were considered statistically significant. Probabilities of survival were calculated using Kaplan–Meier estimates, with differences between the curves based on log-rank tests. Overall survival was defined as the time from diagnosis to the last follow-up or death for any cause. Logistic regression models were applied to investigate the likelihood of association between VISTA expression and the genomic abnormalities. When appropriate, a multivariate model was applied. Linear regression models were used to test the associations between two continuous variables, and the adjusted R-squared was calculated to predict the goodness of fit of these models.

All the analyses were performed using the statistical computing environment R (4.0.0 R Core Team, R Foundation for Statistical Computing, Vienna, Austria).

## Data Availability

All data are accessible through public series or published articles.

## Supplementary Materials

The following supporting information can be downloaded at https://www.mdpi.com/article/10.3390/ijms232314885/s1.
