# Supplementary material for: Comprehensive Transcriptomic Analysis of VISTA in Acute Myeloid Leukemia: Insights into Its Prognostic Value
Source: Int J Mol Sci. 2022 Nov 28;23(23):14885. doi: 10.3390/ijms232314885 (PMC9735915; doi:10.3390/ijms232314885)
Supplement: Supplementary file 1 [file ijms-23-14885-s001.zip › SUPPLEMENTARY_Vista_IJMS_resubmission.pdf]

SUPPLEMENTARY
Comprehensive Transcriptomic Analysis of VISTA in Acute Myeloid Leukemia: Insights into Its Prognostic Value

Simona Pagliuca [1,2], Carmelo Gurnari [1,3], Keman Zhang [1,4], Tariq Kewan [1], Waled Bahaj [1], Minako Mori [1], Ishani Nautiyal [1], Marie Thérèse Rubio [2], Francesca Ferraro [5], Jaroslaw P. Maciejewski [1], Li Wang [1,4] and Valeria Visconte [1,*]

[1] Translational Hematology and Oncology Research Department of Cleveland Clinic, Cleveland, OH 44106, USA
[2] Service d'hématologie, Hôpital Brabois, CHRU Nancy and CNRS UMR 7365 IMoPa, Biopôle de l'Université de Lorraine, 54500 Vandoeuvre les Nancy, France
[3] Department of Biomedicine and Prevention, University of Rome Tor Vergata, 00133 Rome, Italy
[4] Department of Molecular Medicine, Cleveland Clinic Lerner College of Medicine, Case Western Reserve University, Cleveland, OH 44195, USA
[5] Division of Oncology, Department of Medicine, Washington University School of Medicine in St. Louis, St. Louis, MO 63110, USA
* Correspondence: visconv@ccf.org

Description of supplemental material

All the supplementary tables are provided as separate Excel files.

Supplemental tables

Supplemental Table 1: VISTA expression in scRNAseq samples from normal donors

Supplemental Table 2: VISTA expression in hematological cancer cell lines

Supplemental Table 3: Beat AML samples and derived variables used in this study

Supplemental Table 4: Differential gene expression analysis of High VISTA vs Low VISTA expressors

Supplemental Table 5: Upregulated genes in High vs Low VISTA expressors

Supplemental Table 6: Frequency of the top 60 mutated genes in High and Low VISTA expressors

Supplemental Table 7: Vista Expression on *NPM1* mutants from LFR and SFR groups

Supplemental Table 8: Pairwise comparisons of VISTA expression across AML subtypes

Supplemental figures

Figure S1: Detailed distribution of VISTA expression across clinically and biologically defined AML subtypes

Figure S2: Associations between gene mutations and VISTA expression

Figure S3: Relationship between HLA, NPM1 and KRAS and VISTA expression

Figure S4: Disease free survival in NPM1 positive AML subset according to VISTA expression.

Supplementary Figures

Figure S1

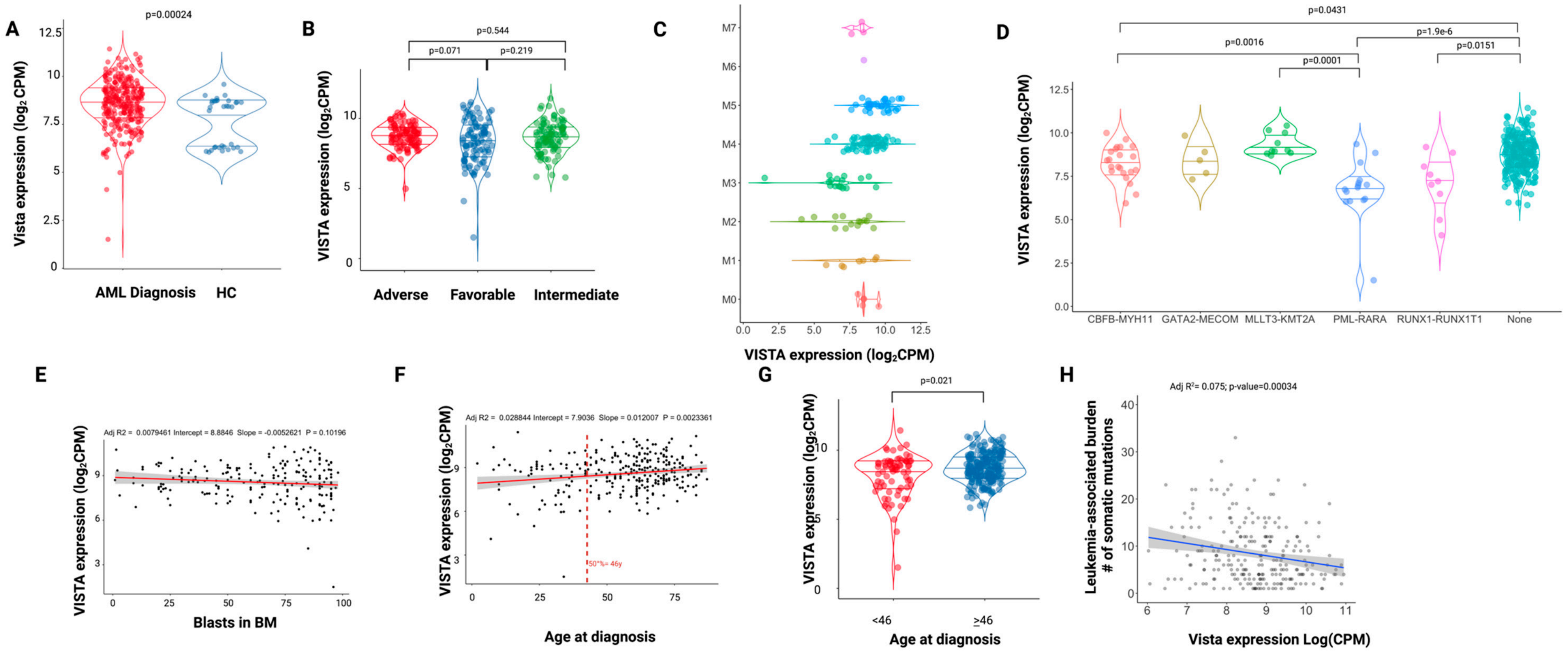

Figure S1: (**A)** Distribution of VISTA expression in AML patients and healthy controls (HC); **B-D**) Distribution of VISTA expression values according to different clinical (B), morphological (C) and genetic subgroups (D) at diagnosis (N=285) . (**E-F**) Linear regression analysis showing the correlations between VISTA expression and blasts in bone marrow (E) and age at diagnosis (F).

(**G**) Distribution of VISTA expression values according to age categories (based on the 50th percentile of age distribution in AML cohort – patients sampled at diagnosis included in the study). (**H**) Linear regression between the number of somatic mutations in AML samples at diagnosis from Beat AML cohort and VISTA expression (N=245).

Figure S2

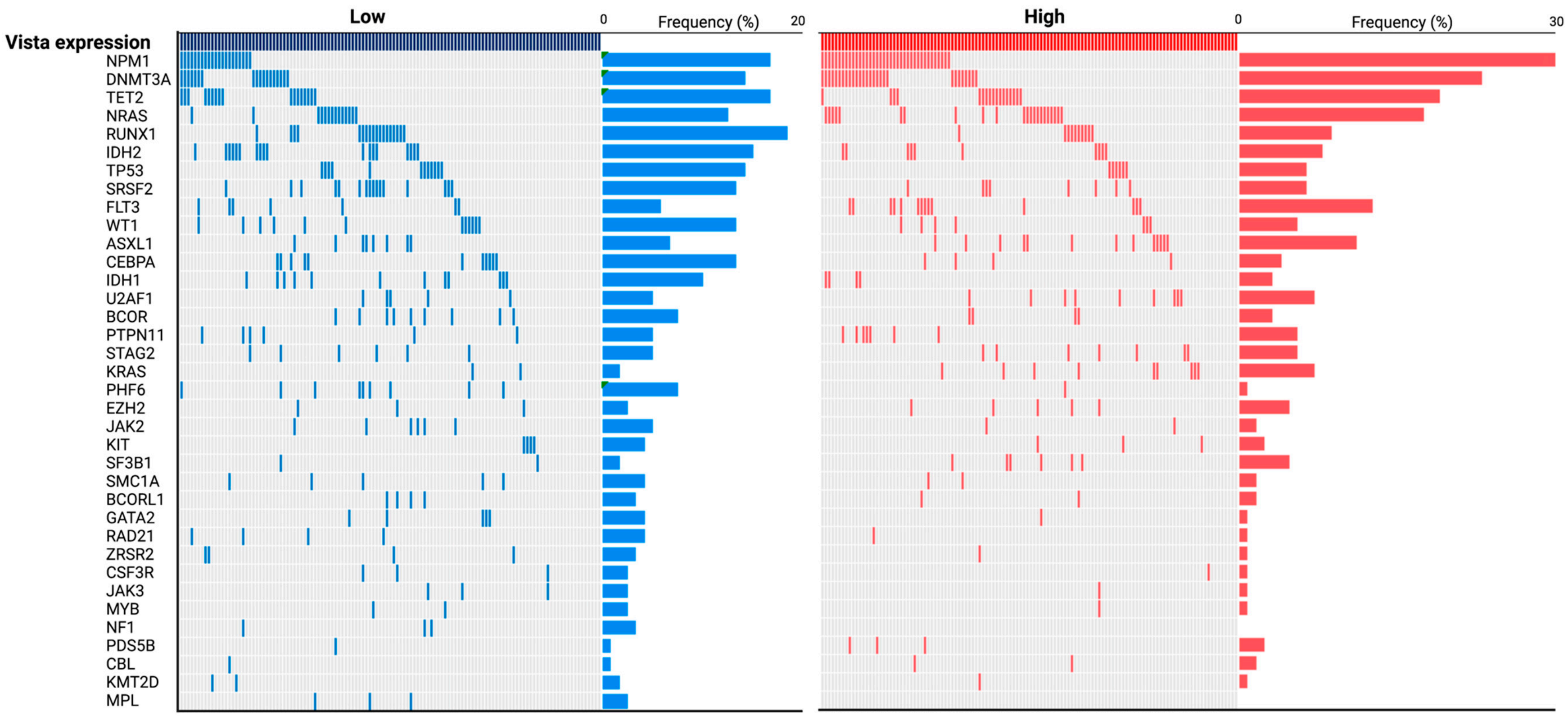

Figure S2: Associations between gene mutations and VISTA expression; Oncoplot showing the distribution of the top 30 mutated genes in low and high VISTA expressors (N=245). The barplot on the right indicates the frequency of each aberration; stars indicate the straightness of the statistical association (Fisher`s exact test; see also Table S7)

Figure S3

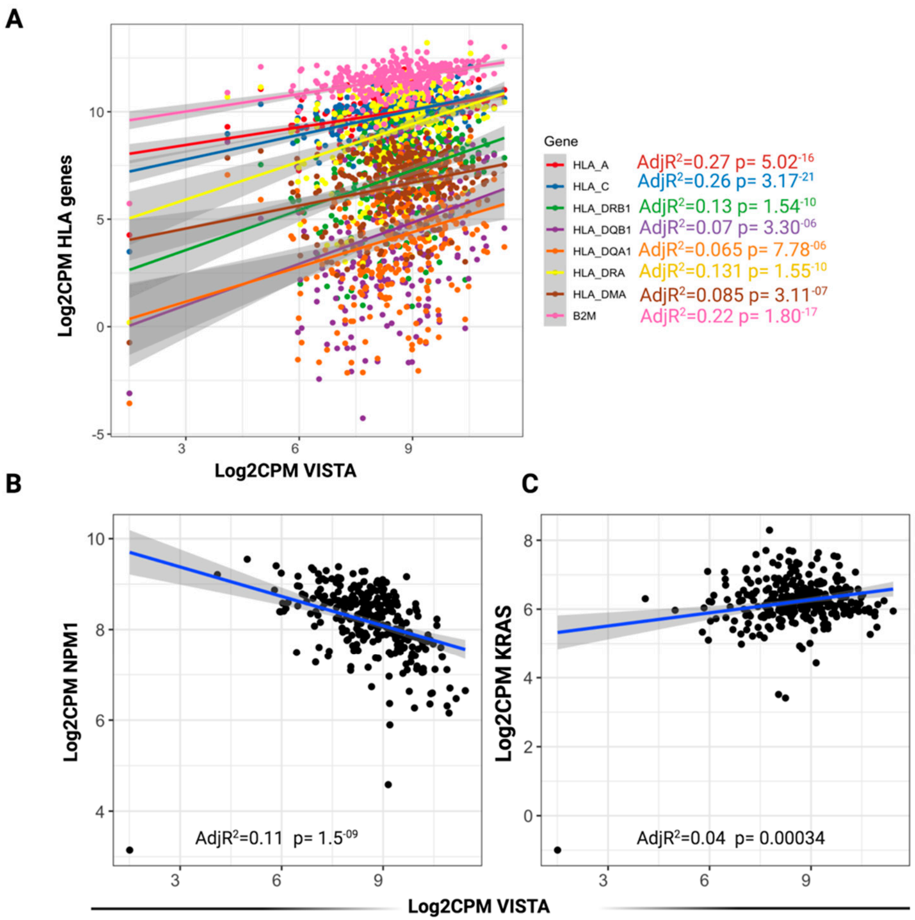

Figure S3: Linear regression between VISTA expression and expression of A) HLA genes and B2M; B) NPM1 C) KRAS in Beat AML cohort (only samples at diagnosis) Expression values are expressed as Log2CPM. Adj R^2 and p values are given for each function.

Figure S4

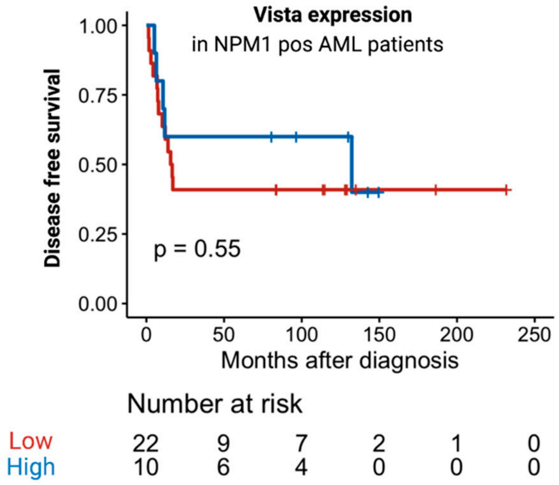

Figure S4: Disease free survival in NPM1 positive AML subset according to VISTA expression (low or high).
